# Key health promotion factors among male members of staff at a higher educational institution: A cross-sectional postal survey

**DOI:** 10.1186/1471-2458-8-58

**Published:** 2008-02-12

**Authors:** Alena Vasianovich, Edwin R van Teijlingen, Garth Reid, Neil W Scott

**Affiliations:** 1 Department of Public Health, Polwarth Building, Medical School, Foresterhill, University of Aberdeen, Aberdeen, AB25 2ZD, UK

## Abstract

**Background:**

Men's lifestyles are generally less healthy than women's. This study identifies associations between health-related behaviour in different groups of men working in a Higher Education (HE) institution. In addition, men were asked whether they regarded their health-related behaviours as a concern. This article highlights smoking, consumption of alcohol and physical activity as most common men's health-related lifestyle behaviours.

**Methods:**

A descriptive cross-sectional survey was conducted among all male staff employed by a Higher Education institute in Scotland using a postal self-completed questionnaire. A total of 1,335 questionnaires were distributed and 501 were returned completed (38% return rate). The data were analysed using SPSS 13.0 for Windows.

**Results:**

Less than 10% currently smoked and almost 44% of these smokers were light smokers. Marital status, job title, consumption of alcohol and physical activity level were the major factors associated with smoking behaviour. Men in manual jobs were far more likely to smoke. Nearly all (90%) consumed alcohol, and almost 37% had more than recommended eight units of alcohol per day at least once a week and 16% had more than 21 units weekly. Younger men reported higher amount of units of alcohol on their heaviest day and per week. Approximately 80% were physically active, but less than 40% met the current Government guidelines for moderate physical activity. Most men wanted to increase their activity level.

**Conclusion:**

There are areas of health-related behaviour, which should be addressed in populations of this kind. Needs assessment could indicate which public health interventions would be most appropriately aimed at this target group. However, the low response rate calls for some caution in interpreting our findings.

## Background

Men's health is poor compared to women's according to a range of measures and varies across ethnicity and socio-economic class [1]. In 2003–05 the average life expectancy at birth of females born in the UK was 80 years compared to about 76 years for males [2]. Men are more likely than women to be mentally ill and they are in greater risk of heart disease and stroke; men in routine and manual jobs are more likely to smoke and have chronic health problems than other men; diagnoses of both prostate and testicular cancer have increased since the early 1990s [1]. The suicide rate amongst young men has increased by 250% over the past two decades [1]. Slightly more than 60% of men are overweight or obese; however, between the ages of 15 and 64 men attend their GP practice almost half as often as women [3]. More generally, men in Scotland have one of the poorest health records in Europe [4]. Life expectancy for Scottish males was the lowest in the four countries of the UK in 2005 (69.3 vs. 79) [5].

Smoking, lack of physical activity and alcohol consumption are among the key lifestyle factors identified in the Government's White Paper as contributing towards poor health and early death [6] and they need to be improved [7]. The latest Scottish Health Survey 2003 (SHS) reported separately the results for men and women [8]. Although, not enough research has been conducted in the field of men's health promotion [9-11].

### Tobacco use

Smoking prevalence amongst men in Scotland is around 28% [12]. Approximately 13,000 Scots die every year from smoking-related illness [7,13,14]. The importance attached by the Government to reducing levels of smoking is emphasised by the publication of two White Papers: 'Smoking Kills', which sets out the actions to be taken throughout the UK to reduce smoking, and 'Towards a Healthier Scotland', which focuses on specific targets for Scotland [15]. There is evidence that male smokers are more likely to be heavy drinkers [16].

### Alcohol use

Alcohol consumption contributes to a wide range of health and social problems, including liver cirrhosis, pancreatitis, cancer, suicide, accidents, and antisocial behaviours [17]. However, alcohol consumption is an established part of Scottish culture, with 27% of men reported usual alcohol consumption in excess of the recommended limit of 21 units per week (a unit refers to half a pint of normal strength beer, a small glass of wine, or a single measure of spirits) [6]. In terms of daily consumption, regular drinking of 4 or more units a day for men is likely to result in increasing health risk and is not advised [6]. There is no significant health risk for adults who regularly consume less than these amounts, though official advice also includes two alcohol-free days a week [6]. Although there is no standard definition of 'binge' drinking it is typically defined as drinking more than double the recommended daily limit on any one day. The Scottish Executive's 'Plan for action on alcohol' stated clearly its overall purpose such as to reduce alcohol-related harm in Scotland [6].

### Physical activity

Levels of physical activity are decreasing [18]. The importance of physical activity and its contribution towards health improvement was recognised by the Government [13]. It sets out plans for a National Physical Activity Strategy for Scotland, to encourage people of all ages to participate more in physical activity. Physical activity protects against a range of diseases including obesity [19]. Men spend a considerable amount of their time working and different jobs/work environments can have different effects on their health and health-related behaviour [20], The World Health Organization (WHO) rated physical inactivity as one of the main causes of death in developed countries, and estimated that it is partly responsible for a range of disease such as coronary heart disease, colon and breast cancer, diabetes and stroke [21]. In Scotland, it has been estimated that an increase of 5% in the proportion of adults participating in physical activity could prevent 157 premature deaths over five years [19]. Taking into account all these evidence, the promotion of physical activity has been described as 'public health's best buy' [6]. The Scottish Executive highlighted the importance of physical activity in improving the nation's health in its 2003 publication 'Improving Health in Scotland – the Challenge' [7]. The current Scottish guideline for physical activity for adults is at least 30 minutes of moderate activity on at least 5 days a week [7]. A questionnaire study on leisure time physical activity, other health-related behaviour, social relationships, and health status showed that persistent physical inactivity is associated with a less healthy lifestyle, worse educational progression, and poor self perceived health [22].

### Men's lifestyle

The choices men make about their behaviour and especially about their consumption of food, alcohol, tobacco and/or physical activity have economic and cultural dimensions. The relationship between socio-economic factors and different health-related behaviour (lifestyle) has long been realised [23]. Despite this, many men need encouragement to consider their own health and to understand the impact of the lifestyle choices they have made [23]. The impact of socio-economic differences on two basic kind of health-related behaviour defined as health behaviour (HB) and risk behaviour (RB) have been illustrated [24]. Risk behaviour refers to behaviour considered to be a risk to health status such as smoking and alcohol consumption. While HB such as physical activity refers to behaviour considered to be health-promoting. HB is demonstrated by individuals considering themselves to be healthy and is directed toward the prevention of illness [23]. Both, health and risk behaviour, have had an association with education, socio-economics status and gender [24].

The importance of gender and its influence on health had led to an increasing interest in gender-specific fields worldwide [25]. The trend has been a move away from basic research on sex and gender differences to new strategies of public health and health promotion, targeting men of all ages and with different risk factors [26]. As some authors summarised [27], men are more likely than women to smoke, drink or use illegal drugs. However, the comparisons made are based on the gender differences between men and women, but these do not explore the differences that may exist among men [27,28]. In the light of the above, the Scottish Government recently invested ˆ4 million into projects to reach men; whose death rates for cancer and coronary diseases are among the highest in the world [29].

This study aims to explore associations between health-related behaviour such as smoking, alcohol use and physical activity, as the most common elements of men's lifestyle, in different groups of men working in Higher Education (HE) on the basis of socio-economics and demographic factors.

## Methods

A cross-sectional survey, using a postal self-completed questionnaire, investigated aspects of men's health-related behaviours [30,31]. The questionnaire is listed as an Appendix (Additional file 1). The survey had a clear descriptive purpose as a way of studying social conditions, relationships and behaviour [32]. The target population was men aged 18 years and over working at a Scottish Higher Educational institution.

Key features of the questionnaire were: health-related behaviour on the basis of having health check-up, blood pressure and cholesterol measurement with knowledge about their level; smoking status and number of cigarettes; alcohol consumption, frequency of drinking and amount of alcohol; physical activity level, having recent stress; health/lifestyle concerns or worries; attitude towards health-related habits by asking participants if they would like to change their habits and possible ways of changing. The questions about alcohol, for example, were taken from the Well Men Services Project questionnaire and included questions about type, frequency and amount of alcohol intake in an average week and on day of highest intake [33]. The number of drinks men consumed was then computed and transformed into units according to existing norms used in the 2003 SHS [8]. The daily benchmark limit for men is four units, where a 25 ml measure of spirits is considered to be a unit, a standard glass of wine is 2 units and a pint of strong lager is three units [8]. We also categorised weekly alcohol intake into three categories based on previous studies: 'safe' (less than or equal to 21 units), 'hazardous' (22–49 units) and 'dangerous' (more than 50 units per week) [8]. For some analyses this variable was transformed into a new binary variable: yes-men who met the current guidelines and consumed less than or equal to 21 units of alcohol per week; no-men who have not met current guidelines for weekly drinking limit (more than 21 units per week).

Smoking status was defined into three categories (non-smoker; ex-smoker; current smoker). This variable was also recoded into a binary variable (smoker vs. non-smoker plus ex-smoker). The number of cigarettes was also categorised on the basis of number of cigarettes smoked per day (light smokers – less or equal to 10; medium smokers 11–15; heavy smokers more than 16). Attitudes towards smoking were presented in three groups: men who would like to keep smoking; those who would like to quit and men who would like to cut down the amount of cigarettes smoked.

Men were asked whether their physical activity meets the current guidelines, which we categorised as: yes – have met the current guidelines and no – have not met [7]. Particularly this variable has been used for the future statistical analysis.

Our questionnaire also included questions about socio-economic and demographic factors such as age, educational level and occupational status. Age was defined in completed years and was used in the statistical analysis to avoid loss of information and statistical power; but a new categorical variable (age banded) was recoded towards to aid additional statistical analysis. To assist the Logistic Regression (LR) analysis some variables had to be recoded. For instance, ethnicity was recoded into two variables: (1) 'White British or European'; and (2) 'Other' that have included Pakistani/Bangladeshi/Black African & Caribbean/any mixed background. We present job title using ten different categories (see Additional file 1), the category 'other' included non-manual jobs such as manager, librarian, research director, or medical illustrator. This variable was also recoded into fewer categories for the LR.

Validated questions were taken from the Scottish Health Survey (SHS) 2003 [8], and the Grampian [34], Liverpool [35] and Tayside [36] lifestyle surveys. In this study most of the questions were close-ended and specifically designed to be analysed, not as individual items of information, but as part of indices which represent general orientations and beliefs expressed in different contexts. Only two questions were open-ended with the respondents invited to reply in their own words and an additional box was provided at the end of the questionnaire for comments. The survey questionnaire was designed "*to collect reliable, valid and unbiased data from a representative sample, in a timely manner and within given resource constraints" *[37]. After piloting [38], 1,344 questionnaires were sent to internal mail addresses of all male members of staff at the HE institution (study participants). Access to them was given through the Personnel Department, which sent out the questionnaire on our behalf to all men working at the university at the time of the study. A written explanation of the study was given in the cover letter and on the questionnaire. Returning the completed questionnaire was taken as consent to participate in study. The anonymous questionnaires were returned to the authors in a pre-addressed envelope.

### Statistical Analysis

Data were analysed using SPSS 13.0 for Windows. The testing of associations was conducted using tests of significance, i.e. parametric (one way t-test, ANOVA) and non-parametric tests (Chi-squared test, Mann-Whitney, Kruskal-Wallis). Univariate analysis (Chi-squared test) was used to explore the associations between categorical variables (lifestyle). Logistic Regression (LR) analysis was used to investigate several variables of interest simultaneously. Associations between lifestyles and socio-economic and demographic factors were evaluated by Odds Ratios (OR) and 95% Confidence Intervals (CI) derived from logistic regression. Several new binary variables were created and recoded before they were entered in the regression model. For all statistical tests a p-value of < 0.05 (shown in bold) was taken to be statistically significant [39].

### Ethical approval

As this study did not include research on NHS patients nor took place on NHS premises no ethical approval was needed from the Local Research Ethics Committee, as the HE in question does not have its own ethical review board no formal ethical approval could be obtained. The questionnaire was approved by the Personnel Department and was piloted before hand. Care has been taken to apply the Helsinki principles of ethical research to this study [40], e.g. the researcher did not have access to the names and addresses of staff and all questionnaires were returned anonymously.

## Results

Five hundred and ten questionnaires were returned; nine were invalid. As a result, 501 were presented for analysis out of 1,335 sent (response rate 38%). The mean (44.9) and median (45.0) age were very similar and the age of respondents ranged from 19 to 68 (SD 11.0). All respondents provided information on marital status. The majority (77 %) was married or lived with a partner. Nearly all (94%) described themselves as White; almost eight out of ten had a university degree, nearly all had a full-time post (89 %), sixty percent had an academic (research/teaching) job title and 74% had a permanent contract. More than half (58 %) had managerial or supervisory responsibilities. Table 1 shows the key demographic and socio-economic factors of respondents.

**Table 1:** Demographic and socio-economic characteristics of men (n = 501)

Factors	number	percentage
**Age**		
24 and under	16	3
25–33	55	11
34–42	131	26
43–50	118	24
51–59	131	26
60 and over	50	10
**Marital status**		
Single	94	19
Married/living with partner	386	77
Separate/divorced	21	4
**Ethnic origin**		
White/British or European	469	94
Asian/Indian/Pakistani/Bangladeshi	9	2
Chinese	8	2
Other South Asian	5	1
Black African	3	< 1
Any mixed background & other	7	1
**Educational/Professional qualifications**		
No qualifications	26	5
Standard/O-grades/GCSEs	19	4
Higher/A levels	9	2
Vocational/Further education	53	11
University undergraduate degree	76	15
University postgraduate degree	318	64
**Job description**		
Full time	447	89
Shift work	18	4
Part time	34	7
**Contract description**		
Permanent	369	74
Short term/less then 1 year	15	3
Fixed term	60	12
1–4 years	51	10
Other	6	1
**Job title**		
Professor/reader	81	16
Lecturer/senior lecturer	127	25
Research fellow	59	12
Research assistant	13	3
Teaching fellow/assistant	22	4
Administrative staff	39	8
Support secretarial	11	2
Support technical	51	10
Support manual	44	9
Other	54	11
**Line management or supervisory responsibilities**		
Yes	292	58

### Smoking

Approximately one in ten, 47 (9 %) respondents currently smoked and 125 (25%) were ex-smokers. There was no statistically significant difference in mean age between smokers, non-smokers and ex-smokers [F (2; 498) = 1.2, p = 0.3]. Current smokers (n = 47) were asked about the number of cigarettes, roll ups or pipes they smoked per day. Twenty men (44%) were light smokers, twelve (24%) were medium smokers and fifteen (32%) were heavy smokers. Younger men (25–33 years) were more likely to be heavy smokers (36%), however, this difference was not statistically significant [χ^2 ^(10) = 15.220; p = 0.12]. Single men (8, 53%) smoked more cigarettes than married men or those living with partner (6, 40%), [χ^2 ^(4) = 9.4, p = 0.05]. According to the LR model, marital status, job title, alcohol consumption and physical activity level were statistically significantly associated with smoking status (Table 2).

**Table 2:** Association between smoking, demographic, socio-economic & lifestyle characteristics (n = 501)

Factors ^(b)^	**Smoking status**				χ^2^-test	Logistic Regression		
					
	smoker (n = 47)		non-smoker (n = 454)^(a)^					
	n	%	n	%	Unadjusted p value	Adjusted p value	OR^(c)^	95% CI

**Age**						0.7		
33 and under	7	10	64	90	0.1	-	1.0	Referent
34–42	12	9	119	91		0.4	1.6	0.5–5.1
43–50	12	10	106	90		0.2	2.4	0.7–8.8
51–59	11	8	120	92		0.5	1.6	0.4–6.3
60 and over	5	10	45	90		0.5	1.6	0.3–8.1
**Marital status**						**0.01**		
Single	15	16	79	84	**0.01**	-	1.0	Referent
Separated/divorced	28	7	358	93		**0.01**	0.3	0.1–0.7
Married/living with partner	4	19	17	81		0.9	0.9	0.2–4.1
**Ethnicity**								
White British or European	46	10	423	90	0.3	-	1.0	Referent
Other	1	3	31	97		0.2	0.3	0.03–2.4
**Education/qualifications**						0.5		
No qualifications	6	23	20	77	**0.001**	-	1.0	Referent
Standard/O grades/GCSEs&Higher/A levels	6	21	22	79		0.9	0.9	0.2–4.1
Vocational/Further education	3	6	50	94		0.3	0.4	0.1–2.5
University undergraduate degree	9	12	67	88		0.5	2.0	0.3–11.4
University postgraduate degree	23	7	295	93		0.7	1.5	0.2–10.1
**Job title**						**0.003**		
Professor/reader	6	7	75	93	**< 0.001**	**-**	1.0	Referent
Lecturer/senior lecturer	7	5	120	95		0.9	0.9	0.2–3.2
Research/teaching fellow/assistant	10	11	84	89		0.4	1.6	0.5–5.8
Administrative, secretarial &support technical	7	7	94	93		0.7	1.3	0.3–6.0
Support manual	15	34	29	66		**0.02**	18.3	2.8–121.4
Other	2	4	52	96		0.7	0.7	0.1–4.6
**Alcohol status (drank less than recommended 21 units per week)**								
Yes	33	8	390	92	**0.01**	-	1.0	Referent
No	14	18	64	82		**0.01**	2.9	1.3–6.4
**Physical activity level**								
Yes (have met guidelines)	14	7	179	93	0.3	-	1.0	Referent
No (have not met guidelines)	33	11	275	89		**0.03**	2.3	1.1–4.9

^(a) ^current smokers vs. non-smokers & ex-smokers.

^(b) ^age, marital status, ethnicity, education, job, alcohol consumption, physical activity level as independent variables.

Men from support manual staff were highly more likely to smoke (OR 18.3; 95% CI 2.8–121.2). Currently smokers were approximately 3 times more likely to consume more than the recommended 21 units of alcohol per week (OR 2.9; 95%CI 1.3–4.9). Those separated/divorced were less likely to smoke (OR 0.9; 95% CI 0.2–4.1). Also, men who do not meet current guidelines for physical activity were more than twice as likely to smoke (OR 2.3; 95%CI 1.1–4.9).

More than half of the current smokers (28, 60%) reported smoking as a problem. Nineteen respondents (40 %) wanted to quit smoking, 15 (32%) wanted to cut down this habit and 13 (28%) wanted to continue smoking. Men who would like to quit smoking were more than nine years younger (mean age 39.4; SD 11.7) than men who did not wish to change smoking behaviour (mean age 48.5; SD 8.1), [F (2; 44) = 4.3, p = 0.020).

### Consumption of alcohol

**Figure 1:**
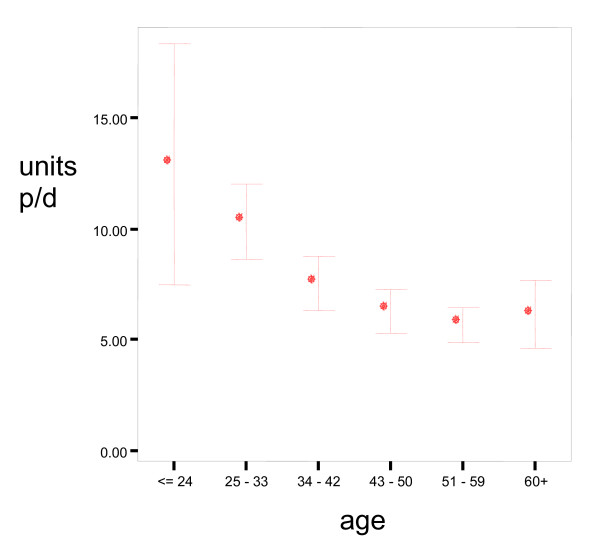
**Units of alcohol per heaviest day and age (n = 49)**. Error Bars show 95.0% confidence intervals (CI) of Mean Dots show Means.

Approximately nine out of ten respondents (n = 449) reported drinking alcohol and they were asked about the frequency of their drinking. The most reported frequency of drinking (37%) was one or two days a week. The most popular drinks for daily and weekly alcohol consumption were alcoholic lemonades (so-called alcopops), beer and wine. Within the other drinks men reported more often sherry or cider. Younger men (≤ 24 years) had more units of alcohol on their heaviest day than relatively older men (51–59 years), [χ^2 ^(5) = 28.9, p < 0.001] as shown in Figure 1.

Heavy drinkers (more than 4 units of alcohol on heaviest drinking day) were more likely to be single, separated or divorced, [χ^2 ^(1) = 5.6, p = 0.02] or without qualifications [χ^2 ^(10) = 20.2, p = 0.03]. The majority (63%) of men on their heaviest drinking day had fewer than 8 units while 37% men had more than 8 units. Average consumption of alcohol on the heaviest day was approximately 7.0 units.

The precise association between alcohol consumption and smoking status is shown in Table 3. The percentage of current smokers was almost twice as high within the group of men who consumed more than the recommended 21 units of alcohol weekly (18% vs. 8%). Also, the percentage of men who have not met the current guidelines for weekly alcohol consumption was higher within the group of ex smokers (32% vs. 23%) and the percentage of men who have never smoked (69%) was higher within the group of men who have met the current guidelines [χ^2 ^(2) = 12.5, p = 0.002]. Current smokers were approximately three times more likely to consume more than the recommended 21 units of alcohol per week (OR 2.8; 95% CI 1.3–6.0) compared with men who currently do not smoke (Table 4).

**Table 3:** Association between alcohol consumption and smoking status (n = 501)

Factors	**Smoking status**								
				
	Current smoker (n = 47)		Ex smoker (n = 125)		Non smoker (n = 329)		Total		Statistical significance
	n	%	n	%	n	%	N	%	p value

**Alcohol status (drank less than recommended 21 units per week)**									

Yes	33	8	100	23	290	69	423	84	
No	14	18	25	32	39	50	78	16	**0.002**

Total	47	9	125	25	329	66	501	100	

**Table 4:** Association between alcohol consumption, some socio-economic and lifestyle characteristics (n = 501)

Factors ^(b)^	Alcohol (drank less than recommended 21 units per week)				χ^2^-test	Logistic Regression		
					
	yes^(a)^, (n = 423)		no (n = 78)					
	n	%	n	%	Unadjusted p value	Adjusted p value	OR	95% CI

**Age**						0.4		
33 and under	53	75	18	25	0.1	-	1.0	Referent
34–42	108	82	23	18		0.8	0.9	0.4–2.0
43–50	105	89	13	11		0.1	0.5	0.2–1.5
51–59	114	87	17	13		0.2	0.5	0.2–1.3
60 and over	43	86	7	14		0.5	0.7	0.2–2.0
**Marital status**						0.1		
Single	71	76	23	24	**0.01**	-	1.0	Referent
Married/living with partner	337	87	49	13		0.3	0.7	0.4–1.4
Separated/divorced	15	71	6	29		0.3	1.9	0.6–6.3
**Ethnicity**								
White British or European	394	84	75	16	0.5	**-**	1.0	Referent
Other	29	91	3	9		0.4	0.6	0.2–2.0
**Smoking status**								
Non smoker&ex smoker	390	86	64	14	**0.01**	-	1.0	Referent
Current smoker	33	70	14	30		**0.01**	2.8	1.3–6.0
**Physical activity level**								
No (have not met current guidelines for physical activity level	272	88	36	12	0.3	-	1.0	Referent
Yes (met current guidelines)	117	92	16	8		0.1	0.7	0.4–1.1
**Job title**						0.06		
Professor/reader	66	82	15	18	**0.04**	-	1.0	Referent
Lecturer/senior lecturer	117	92	10	8		**0.01**	0.3	0.1–0.8
Research/Teaching fellow/assistant	73	78	21	22		0.6	0.8	0.3–1.8
Administrative& Secretarial staff& Support technical	81	80	20	20		0.7	0.8	0.4–1.9
Support manual	39	89	5	11		0.1	0.3	0.1–1.0
Other	47	87	7	13		0.2	0.5	0.2–1.4

^(a) ^have met guidelines for alcohol consumption (≤ 21 unit per week)

^(b) ^age, marital status, ethnicity, job title, smoking status, physical activity level as independent variables.

Separated/divorced men were about twice as likely to drink heavily during the week (OR 1.9; 95% CI 0.4–2.0) compared to single men but this result was not statistically significant (p = 0.1). Men from support manual staff were less likely to have more than the currently recommended units of alcohol weekly (OR 0.3; 95%CI 0.1–1.0) but it was also not statistically significant (p = 0.06) in this study.

Only 27 out of 449 drinkers (6%) reported drinking as a problem and they were more than five years younger (mean age 40.2; SD 11.2) than men who did not report their drinking habit as a problem (mean age 45.2; SD 11.3), [t (446) = 2.3; 95% CI 0.7–9.5; p = 0.02]. Married men were less likely to regard their drinking habit as a problem (95%), [χ^2 ^(2) = 6.6, p = 0.04]. Only 58 out of 449 drinkers (13%) wanted to cut down the amount of alcohol consumed and they were on average younger (mean age 39.6; SD 11.8) than men who responded negatively (mean age 45.7; SD 11.1), [t (446 = 3.9; 95% CI 3.1; 3.9; p < 0.001]

### Physical activity

Over three-quarter of the respondents (77%) reported they were physically active in an average week. However, only 39% met the guidelines for physical activity. Table 5 shows associations between physical activity level, demographic, socio-economic and lifestyle characteristics of men in this study.

**Table 5:** Association between physical activity, demographic, socio-economic & lifestyle characteristics (n = 501)

Factors ^(b)^	**Physical activity level**				χ^2^-test	Logistic Regression		
					
	Yes^(a) ^(n = 193)		No (n = 308)					
	n	%	n	%	Unadjusted p value	Adjusted p value	OR	95% CI

**Age**						0.1		
33 and under	42	59	29	41	**0.001**	-	1.0	Referent
34–42	55	42	76	58		0.2	1.6	0.8–2.9
43–50	36	31	82	69		**0.01**	2.5	1.2–4.9
51–59	44	34	87	66		**0.02**	2.3	1.1–4.7
60 and over	16	32	34	68		**0.03**	2.6	1.1–6.1
**Marital status**						0.3		
Single	49	52	45	47	**0.01**	-	1.0	Referent
Married/living with partner	135	35	251	65		0.2	1.5	0.8–2.4
Separated/divorced	9	43	12	57		0.8	0.9	0.3–2.4
**Smoking status**								
Non smokers&ex smokers	179	39	275	61	0.3	-	1.0	Referent
Current smoker	14	30	33	70		**0.02**	2.4	1.2–5.1
**Alcohol status (drank less than recommended 21 units per week)**								
Yes	156	37	267	63	0.1	-	1.0	Referent
No	37	47	41	53		0.2	0.7	0.4–1.2
**Job title**						**0.04**		
Professor/reader	22	27	59	73	**0.02**		1.0	Referent
Lecturer/senior lecturer	40	32	87	68		0.8	0.9	0.5–1.7
Research/Teaching fellow/assistant	41	44	53	56		0.5	0.8	0.4–1.6
Administrative&Secretarial staff& Support technical	41	41	60	59		0.2	0.7	0.4–1.3
Support manual	23	52	21	48		**0.01**	0.3	0.1–0.7
Other	26	48	28	52		0.1	0.5	0.2–1.1

^(a) ^have met guidelines for physical activity level (30 minutes per day for 5 days a week)

^(b) ^age, marital status, job, alcohol consumption, smoking status as independent variables.

According to logistic regression analysis, smoking status (p = 0.02) and job (p = 0.04) had a statistically significant association with physical activity level. Men from the support manual group were more likely to achieve the current guideline for physical activity (OR 0.3; 95% CI 0.1–0.7) compared with professor/reader. The trend was that physical activity level decreased with the decreasing level of job title. Senior grades such as academic/research took less exercise than junior grades such as support manual. The percentage of men who currently smoke was higher within the group of men with comparatively low levels of physical activity (70% vs. 61%). Within the group of men with adequate levels of physical activity, the percentage of men who described themselves as non-smokers and ex-smokers was higher (39%) than in group of current smokers (30%). More than one third, 38% thought that they did not have enough physical activity. Men with low levels of education (92%) did not have regard their physical activity level to be a problem compared with 44% of men with higher educational achievements [χ^2 ^(5) = 21.3, p = 0.001]. The highest percentage of men who noted that their physical activity level was a problem was in non-manual jobs (55%), [χ^2 ^(10) = 29.5, p = 0.001].

Most respondents wanted to increase their physical activity level (323, 65%) and these were approximately three years younger (mean age 43.9; SD 11.0) than men who did not want to increase it (mean age 46.9; SD 10.8), [t (449) = 2.9; 95% CI 1.0–5.0; p = 0.004]. Both men (70%) with higher educational attainment wanted to increase their physical activity level [χ^2 ^(5) = 18.7, p = 0.002], and the majority in non-manual jobs (75%) reported they wanted more physical activity [χ^2 ^(10) = 21.4, p = 0.02].

## Discussion

In our study relatively few men were current smokers, three times fewer than the Scottish average (9% vs. 29%) [8]. Unlike other studies [8] we did not find an association between age and smoking prevalence. There were fewer heavy smokers in our study compared with the 2003 SHS (33% vs. 38%) [8]. Younger men (25–33) were more likely to be heavy smokers (36%); that is in contrast to the SHS which reported that smokers aged 16 to 34 were much less likely to smoke heavily [8].

The majority consumed alcohol, many (37%) consumed alcohol on 1 to 2 days per week and only 6% consumed alcohol less than once per month. Consuming more than the recommended limit of 21 units per week was reported by approximately 16% of men and this percentage was significantly less than that reported by the SHS (27%) [8]. The average weekly alcohol consumption was again less than reported in the national survey (11.4 vs. 17.2 units) [8]. However, average consumption of alcohol on the heaviest day was similar to the SHS (7.1 vs. 7.4 units) [8]. The Liverpool lifestyle survey reported that 32.9% of men consumed alcohol 1 to 3 times a week and 51.7% consumed alcohol at least once per week with average weekly consumption of 22.6 units [35]. In our survey, only six percent of men reported that their drinking was a problem and it was half of that in the national survey (12%) [8]. Relatively younger men reported drinking as a problem and than was similar to the SHS [8]. In addition, in the current study the percentage of men who have reported drinking as a problem was higher for married men. Younger men were more likely to wish to cut down the amount of alcohol they consumed and married men were more likely to remain at their present level. Multivariate logistic regression was used to examine the factors associated with alcohol consumption. Drinking was statistically significantly associated only with smoking status, whilst the SHS has also reported that drinking is age-related [8]. It is likely that the available statistics on alcohol consumption underestimate the true scale of the situation [41]. The General Household Survey in 2004 found that 65% of people aged over 16 in England had drunk alcohol in the preceding week, of whom nearly half had drunk more than the recommended daily limit on one or more days that week [42]. This English survey also showed that men drank more often than women, and that men were more likely to exceed the daily benchmark quantities of alcohol [42]. The SHS 2003 found that 27% of men and 14% of women typically drank more per week than the recommended limits [8]. Young people drank less frequently but those aged 16 to 24 were more likely than any other age group to have exceeded the daily recommended limits in the previous week [8].

Most (77%) men were physically active in an average week, but less than half (39%) were physically active for at least 30 minutes a day 5 times per week; slightly less compared to the SHS (42%) [8]. Only 27.4% of the adult population in Liverpool achieved moderate physical activity [35]. Smoking status and job title were the only major factors in our study associated with taking the recommended level of physical activity. The SHS reported age, socio-economic status and time spent sitting at a screen [8]. In general, participants in our study reported a relatively high general level of physical activity besides to common risk-behavioural lifestyle factors.

Our response rate was relatively low (38%) compared to the SHS (67%) [8], the Grampian Adult lifestyle survey (52.6%) [34], Tayside lifestyle survey (61%) [36], or the Liverpool lifestyle survey (39.9%) [35]. This might be related to the specific focus on men's health, sampling technique that has been used and/or the target groups of busy HE staff. Men often struggle with balancing a dilemma between 'do not care' and 'should care' [43], which may also influence their decision to participate in men's health research. Hence, it would be important to find out ways of increasing the response rate and carry out qualitative research in future studies to identify the causes of concerns and how it affects men's health seeking behaviour.

## Conclusion

Limitations of our study were (1) its cross-sectional nature; (2) the response rate; and (3) not every area of life, lifestyle and health-related behaviour was covered in the questionnaire [44]. The study size was not calculated since the Personnel Department posted questionnaire to all men (100%) and sample size was fixed. As was to be expected in a HE population, men had high educational attainment (78%) and the majority were in non-manual jobs (60%). As HE institution staff, they were better educated than the general population and had better knowledge of health-related behaviour but they still showed unhealthy lifestyle behaviours. Our findings might suggest that many would benefit from a health promotion intervention offering advice, support and involvement in physical activity or behaviour changes. HE institutions, as potentially health-promoting workplaces, may help to encourage staff to change lifestyles [45]. A few risk factors were more common in this sample than in the general population, which would suggest that some health promotion intervention such as to change behaviour aimed at men working in HE might be appropriate.

The increased interest in men's health promotion over the past decades has been mirrored by a theoretical development around concepts of masculinity [46]. Gender differences affect both health and illness and the way men and women think, feel and behave [47]. Therefore, some have argued that men need to be targeted especially in public health prevention campaigns [48]. In spite of some shortcomings, our study shows that there are areas of health-related behaviour, which should be addressed to this study population. Hence, there appears to be scope for public health interventions aimed at this target group. It might be worth considering changes in the work environment and/or behavioural-change approaches to help men to adopt healthy behaviours.

## Abbreviations

HE, Higher Education; HB, Health Behaviour; RB, Risk Behaviour; OR, Odd Ratio; CI, Confidence Interval; GSCE, General Certificate of Secondary Education; LR, Logistic Regression; SHS Scottish Health Survey; SD, Standard Deviation, UK, United Kingdom, WHO World Health Organisation.

## Competing interests

The author(s) declare that they have no competing interests.

## Authors' contributions

EvT, AV and GR were responsible for the study conception and design and drafting of the manuscript. AV performed the data collection, AV conducted the data analysis. EvT, AV, GR and NS made critical revisions to the paper. All authors read and approved the final manuscript.

## Pre-publication history

The pre-publication history for this paper can be accessed here:



## Supplementary Material

Additional file 1 Men's Health Questionnaire. Copy of the questionnaire designed for and used in our men's health study. Click here for file
