# Supplementary material for: Key health promotion factors among male members of staff at a higher educational institution: A cross-sectional postal survey
Source: BMC Public Health. 2008 Feb 12;8:58. doi: 10.1186/1471-2458-8-58 (PMC2270269; doi:10.1186/1471-2458-8-58)
Supplement: Additional file 1 — Men's Health Questionnaire. Copy of the questionnaire designed for and used in our men's health study. [file 1471-2458-8-58-S1.doc]

# Men’s Health Questionnaire

**Section 1.**  Years

1. **Age**…………………………………………………………………………………………..

e.g. A B 8 8 - 1

**2. Postcode (but not the last two digits**…………………………….

**3. Which statement best describes you at present?**...... (TICK ONLY ONE BOX)

Single…………………………………….. Separated/Divorced…………………………..

Married/Living with partner…………….. Widowed..………………………………………

**4. Ethnic Origin** .…..(TICK ONLY ONE BOX)

White /British/European or other …….. Black Caribbean………………………………

Asian /Indian/Pakistani/Bangladeshi…. Black African…………………………………..

Chinese………………………………….. Any mixed background………………………

Other South Asian……………………… Other ethnic groups………………………….

**5. Educational/Professional Qualifications** ……(PLEASE TICK HIGHEST LEVEL REACHED)

No qualifications………………………… Vocational/Further education……………….

Standard grades/“O”grades/GCSE’s/ University undergraduate degree…………..

Higher/A levels …………………………. University postgraduate degree…………….

**6. How would you describe your job?** …… (TICK AS MANY AS APPLY)

Full time …………………………………. Part time……………………………………….

Shift work ……………………………….. Officially on call……………………………….

**7. How would you describe your contract?**......(TICK AS MANY AS APPLY)

Permanent………………………………. Fixed Term…………………………………….

Short term/ less then 1 year…………… 1 – 4 years … Other………….

**8.a. What is your job title?**...... (TICK ONLY ONE BOX)

Professor/Reader……………………….. Administrative Staff………………………….

Lecturer/Senior Lecturer………………. Support Staff – Secretarial…………………

Researcher Fellow……………………… – Technical…………………..

Research Assistant…………………….. – Manual…………………….

Teaching Fellow………………………… Other…………………………………………

Teaching Assistant …………………….. Please specify…

**8.b. Do you have any line management or supervisory responsibilities?** (TICK ONLY ONE BOX)

Yes No

**Section 2.**

**9.a. Have you had your blood pressure measured**

**in the last 2 years?**…….………………………….… Yes No . Don’t know

**9.b. If yes, was it normal?** ……………………………… Yes … No Don’t know

**10.a. Have you had your cholesterol measured**

**in the last 2 years?**……………………………... .. Yes No .. Don’t know

**10.b. If yes, was it normal? ( less than**

**5.0mmol/l)?**………………………………………. .. Yes No Don’t know

**11. Have you had a health check in the last 2 years?** Yes No Don’t know

**12. How is your physical health in general? Would you say it is:**

(TICK MOST APPROPRIATE FACE)

|  | 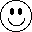 |  | | 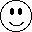 |  | | | 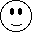 |  | | | 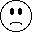 |  | | 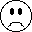 |  |
| --- | --- | --- | --- | --- | --- | --- | --- | --- | --- | --- | --- | --- | --- | --- | --- | --- |
| Very Good | | | Good | | |  | Fair | | |  | Bad | | | Very Bad | | |

**13. How is your mental health in general? Would you say it is:** (TICK MOST APPROPRIATE FACE)

|  | 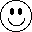 |  | | 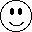 |  | | | 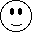 |  | | | 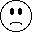 |  | | 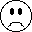 |  |
| --- | --- | --- | --- | --- | --- | --- | --- | --- | --- | --- | --- | --- | --- | --- | --- | --- |
| Very Good | | | Good | | |  | Fair | | |  | Bad | | | Very Bad | | |

**14. Do you have any health/lifestyle concerns/worries?** (PLEASE TICK ALL THAT APPLY)

Diet………………………….. Long standing health problem (e.g. arthritis**)**…………..

Weight………………………. Relationships………………………………………………

Stress……………………….. Anger/aggression ………………………………………..

Alcohol intake……………… Sexual health………………………………………………

Smoking level……………… Mental health………………………………………………

Physical activity levels……. Employment……………………………………………….

Substance abuse………….. Income/debt……………………………………………….

Domestic abuse……………. Other Specify:

Recent illness (e.g. flu)…….

**15. Have you consulted a doctor about any of these concerns/worries?**……. Yes No

**16. Would you like more information on any of these issues?** ……………… Yes No

**17. If yes, which issues would you like more information about?**……..

**18. Do you have any long-standing illness, disability or infirmity?**…………… Yes No

(By long standing we mean anything that has troubled you over a

period of time, or that is likely to affect you more than a year)

**19. Have you ever regularly smoked cigarettes/rolled up cigarettes, a pipe or cigars?**

(TICK ONE BOX)

Never smoke………………………… ………GOTO Q 23

Ex smoker…………………………… ……..GOTO Q 23

Current smoker………………………. ……..GOTO Q 20

Number

**20. How many cigarettes/ rolled up cigarettes, a pipe or cigars do you smoke per day?**

**21 Do you think your smoking is a problem?**…………………………………….. Yes No

**22. Do you want to:** …………......................................…..Keep up smoking Quit Cut down

**23. Do you drink alcohol?**……………. Yes No …… IF NO GOTO Q 29

**24. How often do you drink alcohol?** …………(TICK MOST APPROPRIATE BOX)

Less than once a month……………………………………………………………………………..

More than once a month but not weekly……………………………………………………………

1-2 days per week……………………………………………………………………………………

3-5 days per week…………………………………………………………………………………….

6-7 days per week…………………………………………………………………………………….

**25. How many alcoholic drinks do you have in a WEEK?**

Number

Pints/cans of normal beer/lager……………………………………………………………..

Bottles of normal beer/lager………………………………………………………………….

Shots of spirits…………………………………………………………………………………

Small glasses of wine…………………………………………………………………………

Bottles of alcopops……………………………………………………………………………

Other drink (a) specify…………………………………………………………………………..

Other drink (b) specify…………………………………………………………………………..

Other drink (c) specify…………………………………………………………………………...

**26. How many alcoholic drinks do you have on your heaviest drinking DAY?**

Number

Pints/cans of normal beer/lager………………………………………………………… …..

Bottles of normal beer/lager…………………………………………………………….. …..

Shots of spirits………………………………………………………………………………….

Small glasses of wine………………………………………………………………………….

Bottles of alcopops…………………………………………………………………………….

Other drink (a) specify…………………………………………………………………………..

Other drink (b) specify…………………………………………………………………………..

Other drink (c) specify………………………………………………………………………..

**27. Do you think your drinking is a problem?**……………………….. Yes No

**28. Do you want to cut down the amount of alcohol you drink?**….. Yes No

**29. Have you used illegal drugs during the last year?**………….……Yes No

**30. Have you ever used illegal drugs?**………………………………….Yes No

**31. If yes, what kind of illegal drugs you have used?**……

**32. In an average week, are you physically active?**…………………. Yes No

(By physically active we mean anything that made you hot or breathless or sweaty)

**33. Do you do this activity for least 30 minutes a day**

**5 times per week?**…………………..………………………………… Yes No

(You did not need to do it all at once, e.g. you could have three ten minute bursts of activity in one day)

**34. Do you think your level of physically activity is a problem?**….. Yes No

**35. Do you want to increase your physical activity levels?**…………. Yes No

**36. Have you recently** (TICK ONE BOX PER LINE)

at home at work both neither

1. felt under pressure or stress?…….………………………………………………………………
2. felt you are losing patience, becoming tense/irritated with

people and things?....................................................……………………………………………….

**37. Compared with this time last year do you** (TICK ONE BOX FOR EACH OPTION)**:**

a. feel as secure in your job?………………………………………………. Yes No

b. think about work more during your leisure time?…………………….. Yes No

c. take more work home?………………………………………………….. Yes No

d. miss lunch more often?……….………………………………………… Yes No

e. feel a loss of sense of identity?………………………………………….. Yes No

1. feel less confident in yourself ?................………………………………Yes No

1. worry over the health or other problems of members of

your family?………………………………………………………………..Yes No

h. lose much sleep over worry?……………………………………………..Yes No

i. feel you can not overcome your difficulties?…………………………… Yes No

j. feel constantly under strain?…………………………………………….. Yes No

**38. When you feel under pressure or stress how do you deal with this? Do you:**

(TICK ONE BOX FOR EACH OPTION)**:**

1. try to work out a solution?……………………………………….……… Yes No

b. ignore the problem?………………………………………………. ……. Yes No

c. stay away from others and keeping your feelings to yourself?……. ...Yes No

d. spend more sociable time, going out with friends or family?………… Yes No

e. work harder?……………………………………………………………… Yes No

f. talk to someone about the problem?…………………………………… Yes No

39. Please use the space below to write any comments you have about this questionnaire.
